# Consequences of Decreased Light Harvesting Capability on Photosystem II Function in *Synechocystis* sp. PCC 6803

**DOI:** 10.3390/life4040903

**Published:** 2014-12-11

**Authors:** Aparna Nagarajan, Lawrence E. Page, Michelle Liberton, Himadri B. Pakrasi

**Affiliations:** 1 Department of Biology, Washington University, St. Louis, MO 63130, USA; E-Mails: aparna@biology2.wustl.edu (A.N.); lawrenceepage@gmail.com (L.E.P.); mliberton@biology2.wustl.edu (M.L.); 2 Terra Biologics, St. Louis, MO 63132, USA

**Keywords:** phycobilisome, antenna, photosynthesis, photosystem I, photosystem II, oxygen evolution

## Abstract

Cyanobacteria use large pigment-protein complexes called phycobilisomes to harvest light energy primarily for photosystem II (PSII). We used a series of mutants with partial to complete reduction of phycobilisomes to examine the effects of antenna truncation on photosystem function in *Synechocystis* sp. PCC 6803. The antenna mutants CB, CK, and PAL expressed increasing levels of functional PSII centers to compensate for the loss of phycobilisomes, with a concomitant decrease in photosystem I (PSI). This increased PSII titer led to progressively higher oxygen evolution rates on a per chlorophyll basis. The mutants also exhibited impaired S-state transition profiles for oxygen evolution. Additionally, P700^+^ re-reduction rates were impacted by antenna reduction. Thus, a decrease in antenna size resulted in overall physiological changes in light harvesting and delivery to PSII as well as changes in downstream electron transfer to PSI.

## 1. Introduction

Photosynthesis begins with the capture of a photon by a pigment. Photosynthetic organisms contain pigment proteins organized into antenna, which are highly diverse in structure among different organisms but in all cases function to greatly improve light harvesting capability. In cyanobacteria, a major light-harvesting antenna is the phycobilisome, which associates with the thylakoid membrane and facilitates the absorption and transfer of light energy to the photosynthetic reaction centers [1]. Phycobilisomes preferentially harvest light for photosystem II (PSII), but can also harvest light for photosystem I (PSI) through a mechanism known as state transition when the excitation balance between the photosystems is unequal [2]. Recent work has revealed a functional megacomplex containing phycobilisome, PSI, and PSII that is proposed to represent the spatial organization of these complexes in the thylakoid membrane system [3].

Modifications to light harvesting antenna in photosynthetic organisms for the purpose of increasing productivity have been a topic of considerable interest [4,5]. In the unicellular model cyanobacterium *Synechocystis* sp. PCC 6803 (hereafter *Synechocystis* 6803), the phycobilisome is a hemidiscoidal complex made up of a tricylindrical allophycocyanin core from which six phycocyanin rods radiate [6,7]. A set of antenna reduction mutants has been generated in this cyanobacterium that includes partial and total phycobilisome reduction strains harboring mutations that effectively reduce the pigment and protein content by disrupting the structural genes necessary for phycobilisome synthesis and assembly [8,9]. The CB mutant has phycobilisomes with rods containing only one phycocyanin hexamer, the CK mutant lacks phycocyanin rods completely, containing only the allophycocyanin core, and the PAL mutant cannot assemble any functional phycobilisomes.

Our previous analyses of these mutants showed altered thylakoid membrane organization and changes in photosystem stoichiometry and localization that were progressively more severe with the degree of phycobilisome truncation [10,11]. Furthermore, the reduction in size of phycobilisomes was found to decrease photoautotrophic productivity in a variety of settings [12]. However, the mechanism causing the decreased growth and biomass accumulation in the mutants remained poorly understood. In the current work we have used the same series of antenna truncation mutants to examine the effects of antenna truncation on photosystem function. Levels of both photosystems were evaluated by changes in the ratio of PSI to PSII, and the amounts of functional PSI and PSII in each strain were determined. Our results indicate that the antenna mutants express increasing levels of functional PSII to compensate for the loss of phycobilisomes, and that this increased PSII titer leads to progressively higher oxygen evolution rates. These mutants also demonstrate increased misses in S-state transitions consistent with a decreased antenna cross section per reaction center. P700^+^ reduction kinetics also showed differences in the rates of electron transfer from PSII to PSI. The loss of optimal function of PSII and PSI likely caused the reduction in photoautotrophic productivity observed previously [12].

## 2. Experimental Section

### 2.1. Strains and Growth Conditions

Cyanobacterial strains were routinely maintained on solid BG11 plates [13] with antibiotic selection (CB and CK, 10 µg/mL kanamycin; PAL, 10 µg/mL chloramphenicol and spectinomycin) at 30 °C under constant 30 µmol photons m^−2^·s^−1^ white fluorescent light. Liquid cultures were grown at 30 °C under 30 µmol photons m^−2^·s^−1^ light with appropriate antibiotics, and for use in measurements were subcultured at 10% volume without antibiotics. Cultures grown to exponential phase were harvested and normalized to equal chlorophyll (Chl) concentrations by methanol extraction and measuring absorbance on an Olis DW 2000 spectrophotometer (SLM-Aminco, Urbana, IL, USA) [14].

### 2.2. Low-Temperature Fluorescence Spectroscopy

Fluorescence emission spectra of whole cells were determined at 77 K on a Fluoromax-2 fluorometer (Jobin Yvon, Cedex, France). Chlorophyll was excited at 435 nm and phycobilins were excited at 600 nm [15]. Fluorescence emission spectra were normalized by F/F_750_.

### 2.3. PSI Measurements

P700 concentration in whole cells was determined using a JTS-10 pump probe spectrometer (BioLogic, France). Cultures grown to mid-exponential phase were normalized to 2.5 μg/mL Chl and incubated at 30 °C under 30 μmol photons m^−2^·s^−1^ light prior to measurements. For quantification of P700, samples were dark adapted for 3 min before the actinic light was turned on and optical changes at 705 nm were monitored during 5 s of illumination. The molar extinction coefficient used in this analysis for P700 (70,000 M^−1^·cm^−1^) was estimated from previous report [16]. Maximal changes in the oxidation state of P700 were used to calculate the PSI concentration in WT, CB, CK, and PAL cells.

### 2.4. Steady State Oxygen Evolution Measurements

Photosystem II-mediated oxygen evolution was measured in whole cells on a Clark-type oxygen electrode [17]. Cells were normalized to 5 µg/mL Chl and incubated at 30 °C under 30 μmol photons m^−2^·s^−1^ light. For measurements of PSII-mediated O_2_ evolution, 0.5 mM 2,6-dichloro-*p*-benzoquinone (DCBQ) (Eastman Kodak Co., Rochester, NY, USA) and 1 mM K_3_Fe(CN)_6_ (Sigma Aldrich, St. Louis, MO, USA) were added to samples. Oxygen evolution rate at a given light intensity was measured in triplicate. Curves for calculation of K_m_ and V_max_ were fitted using Kaleidagraph (Synergy Software, Reading, PA, USA).

### 2.5. Western Blots

Total membranes were prepared as in [18]. Proteins from whole membrane preparations were loaded at 2 µg Chl and fractionated by using a resolving gel with 12% acrylamide and 6 M urea. After transfer to 0.45 μm PVDF, CP43 and PsaA were detected by using antiserum against each protein, and both were reacted with goat anti-rabbit horseradish peroxidase conjugated antiserum (Pierce Biotech, Rockford, IL, USA). Blots were developed using Immobilon Western chemiluminescent HRP substrate (Millipore, Billerica, MA, USA) for 2 min and visualized on a Fujifilm LAS-1000 plus imager (Fujifilm, Stamford, CT, USA) for 0.5 to 3 min.

### 2.6. Flash-Induced Oxygen Evolution

Oxygen yield was measured as described previously [19]. Cells were normalized to 40 µg/mL Chl, centrifuged at 16,000× g for 5 min, and applied to a bare platinum electrode (Artisan Scientific Co., Urbana, IL, USA) as a paste. Cells were dark-adapted for 2 min before electrode polarization at 0.65 V for 10 s. A series of flashes were supplied by an integrated, computer-controlled Xenon flash lamp (20 ms width at ½ height). Twenty flashes were applied at 200 ms intervals. The collected data were processed by distilling out maximum peaks using in-house-developed software. Peak data were fitted to a four-step homogenous model for Mn_4_CaO_5_ cluster S-state cycling [20]. Calculations were carried out using MathCad software (MathSoft Engineering and Education, Inc., Cambridge, MA, USA).

## 3. Results

### 3.1. Low-Temperature Fluorescence

**Figure 1:**
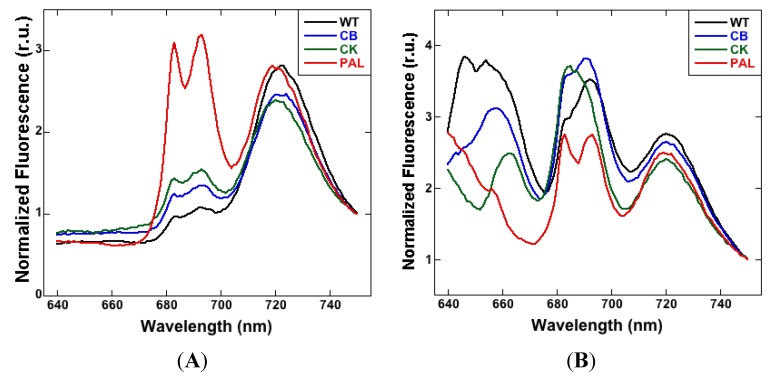
Low temperature (77 K) fluorescence emission spectra recorded with excitation at 435 nm (**A**) and 600 nm (**B**). Samples were loaded at equal chlorophyll concentration (5 μg/mL). Spectra were normalized by F/F_750_ to determine relative changes in PSII and PSI.

When Chl *a* is excited at 435 nm, the normalized fluorescence emission spectra showed evidence of an increase in the PSII:PSI ratio in the mutant strains (Figure 1A). The PSII peaks at 685 nm and 695 nm gradually increased over WT in CB and CK. In PAL, they were significantly higher than in WT, as previously observed [21].

By exciting PBSs at 600 nm, energy transfer to the photosystems was tracked (Figure 1B), and the phenotypic differences in the PBSs of mutants became apparent. The PC emission signal at 645 nm was much smaller in CB, and absent in CK and PAL. The APC peak at 660 nm was present in WT, CB, and CK, but missing in PAL. Changes in the protein environment around PSII in antenna mutants were evident from the differences in the two PSII peaks (685 nm and 695 nm). In WT and CB, the 695 nm peak is higher than the 685 nm peak. In CK, the 685 peak is higher, and in PAL, the two peaks are approximately equal. These results can be interpreted to mean that proximal antenna proteins play a role in energy transfer to the PSII reaction center to different degrees in the mutants, as discussed below.

### 3.2. Photosystem Stoichiometry and PSI Quantitation

**Figure 2:**
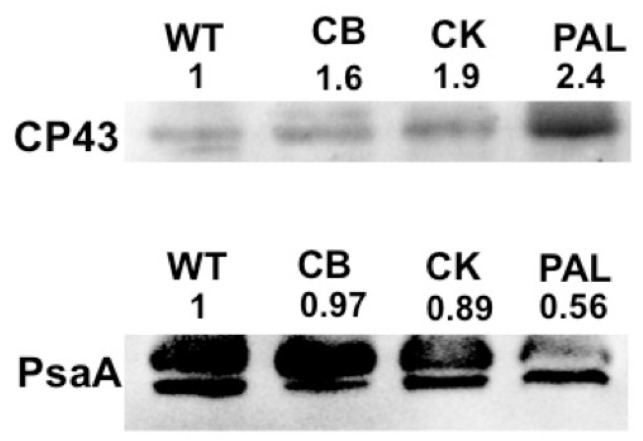
Immunoblot detection of PSII (CP43) and PSI (PsaA) to quantitate photosystem titers. Total membranes from WT, CB, CK and PAL were loaded at 2 μg Chl. Each lane was quantified using Image J and values represent changes relative to WT.

Levels of PSI and PSII were estimated in the antenna truncation mutants by immunoblot analyses with antibodies against PsaA and CP43, respectively (Figure 2). When loaded at equal Chl concentrations, PSII titers in the antenna mutants gradually increased as PBSs were truncated. This increase, however, was not as pronounced in WT, CB, and CK, which can be grouped together with similar PSII content. PAL, on the other hand, showed more than twice the amount of PSII compared to WT. A decrease in PSI was observed as the antenna size decreased, with WT, CB, and CK showing similar PSI content. PAL showed a 50% decrease in PSI when compared to WT. These data suggest a similar trend as observed in Figure 1A when mutants were excited at 435 nm for Chl *a* fluorescence emission. These changes in stoichiometry confirmed our earlier observation made from spectral data of PSII and PSI by hyperspectral confocal fluorescence microscopy (HCFM) [10].

**Figure 3:**
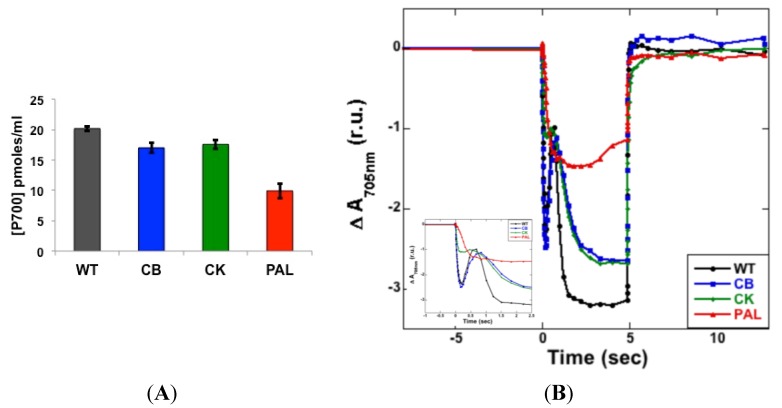
P700 oxidation and reduction measured on a JTS-10 pump probe spectrometer. (**A**) Concentration of PSI measured based on maximum photo-oxidizable P700. Samples were adjusted to a Chl concentration of 2.5 μg/mL and error bars represent standard deviation of four replicates; (**B**) P700^+^ kinetics measured by monitoring changes in absorbance at 705 nm during a 5 s illumination with orange light. Traces represent relative changes in oxidation (downward deflection) when actinic light was turned on and re-reduction (upward deflection) when light was turned off. The inset shows the differences in rate of oxidation two seconds after the actinic light is turned on.

The concentration of PSI was measured by monitoring optical changes at 705 nm post-illumination in all strains. A decrease in maximum P700 available for oxidation was observed with the reduction in antenna size (Figure 3A). PAL showed a 50% decrease (10 pmol P700/mL) in PSI content compared to WT with 20 pmol P700/mL. This decrease was consistent with the observed decrease in immunoblots (Figure 2). PSI concentration in CB and CK were similar with 17 and 17.6 pmol P700/mL, respectively, slightly lower when compared to WT.

The kinetics of P700 oxidation revealed considerable differences between the different antenna truncation mutants (Figure 3B). In WT and CB, a transient reduction of P700 was observed in 0.5 s as a rise after the initial oxidation, possibly due to transfer of electrons originating from PSII to P700^+^ (Figure 3B inset). CK and PAL lacked this reduction, suggesting an inefficient reduction of the PQ pool in the absence of PC rods. PAL was also observed to have a slow reduction of P700^+^ after the actinic light was turned off when compared to other strains. A difference in the maximum ΔA_705nm_ observed in mutants was indicative of the different PSI concentrations, as shown in Figure 3A.

### 3.3. Measurement of PSII Activity

**Figure 4:**
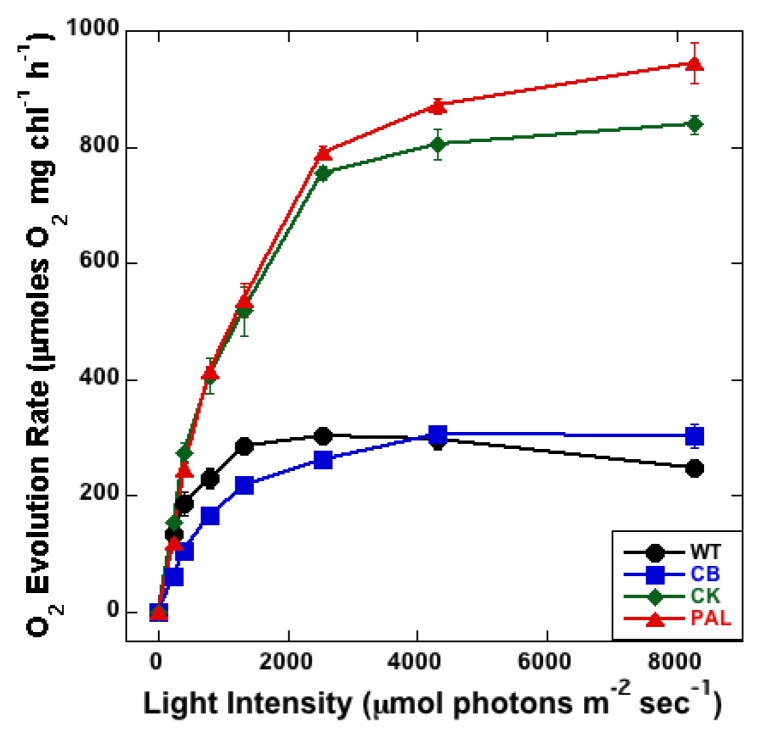
PSII-mediated light saturation curves measured on a Clark-type oxygen electrode in the presence of K_3_Fe(CN)_6_ and DCBQ. Error bars represents standard deviation of three technical replicates.

Light-saturated oxygen-evolving capability was evaluated for antenna mutants using a Clark-type electrode. Increased oxygen evolution was observed with the increase in PSII reaction center titer as the antenna mutants evolve progressively more oxygen per Chl (Figure 4). This correlated with the increase in PSII titer as seen in western blots (Figure 2). WT and CB were observed to have similar rates of maximum oxygen evolution at 8250 µmol photons m^−2^·s^−1^. A large increase in oxygen evolution was observed for CK compared to CB, whereas CK and PAL were observed to have maximum rates of oxygen evolution that were much higher than those measured for WT and CB. The increased rates in CK have been observed previously [22]; however, we measured a considerably larger increase in oxygen evolution in CK compared to WT than was seen in the earlier study (4-fold increase *vs.* 1.25-fold increase). When steady state oxygen evolution rates were fitted to Michaelis–Menten kinetics, both K_m_ and V_max_ were found to increase as antenna are truncated (Table 1). However, the increase in both parameters followed different trends. K_m_ showed a large increase between WT and CB, indicative of the loss of ability for light capture by PSII due to truncation of PC rods. The increased V_max_ values in CK and PAL compared to WT and CB are reflective of the increased PSII titer as well as the decreased ability for PSII to capture light due to the absence of PC rods and APC core.

### 3.4. Flash-Induced Oxygen Evolution

**Figure 5:**
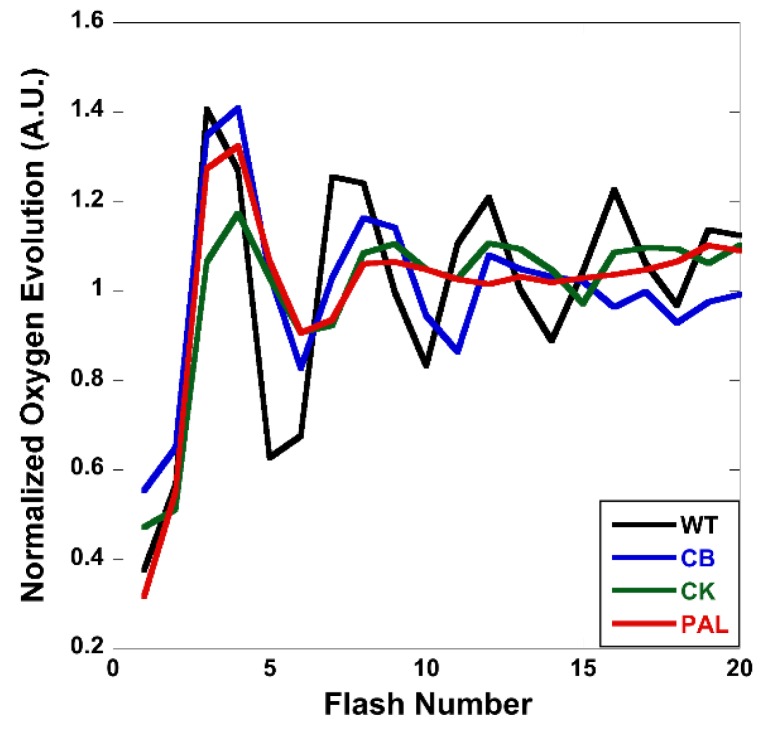
Flash-induced oxygen evolution measured on a bare platinum electrode polarized at 650 mV. Each trace is an average of three technical replicates normalized to the average oxygen evolution rate.

A series of saturating flashes were used to evaluate the period four oscillatory pattern described as the S-state cycle in the antenna truncation mutants. The S-state transitions reflect the accumulation of oxidizing equivalents by the Mn_4_CaO_5_ cluster during oxygen evolution [23,24]. All four strains showed the ability to evolve oxygen on the third and fourth flash (Figure 5). The antenna mutants also evolved more oxygen on average than WT, when data was not normalized to average oxygen evolution (data not shown). However, the damping rate was significantly increased in the antenna mutants. CB and CK were significantly more damped than WT, but still showed cyclic oxygen evolution to the twentieth flash. In PAL, damping was extreme, so severe as to flatten oxygen evolution to the average by the eighth flash.

In fitting the data, the antenna mutants showed similar calculated S-state distributions to WT (Table 1). The probability of a single hit occurring decreases as antenna size decreases, with minor differences between WT and CB (4.4%) and CK and PAL (1.3%). A significant (12.3%) increase occurs between CB and CK. Concurrently, misses increase as antenna size decreases, with a similarly large increase seen between CB and CK (10.1%). Here, WT and CB group together, as do CK and PAL. A similar difficulty in charge delivery to downstream acceptors in PSII was observed when a dual modulated kinetic fluorometer was used to measure flash-induced chlorophyll fluorescence (data not shown).

**Table 1:** Changes in photosynthetic performance of antenna mutants. K_m_ and V_max_ were calculated from PSII-mediated oxygen evolution rates. S-state distribution was calculated from a homogenous four-step mechanism for Mn_4_CaO_5_ cluster cycling using MathCad software.

	WT	CB	CK	PAL
K_m_	302.3	1345.2	1426.2	2864.5
V_max_	312.5	416.7	1111.1	1666.7
S0	34.9	33.7	36.3	31.7
S1	42.5	39.1	40.5	49.3
S2	12.8	14.6	10.7	11.6
S3	9.8	12.6	12.4	7.4
Misses	10.6	14.3	24.4	24.9
Single Hits	86.7	82.3	70.0	68.7
Double Hits	4.9	−0.9	3.0	2.6
Deactivation	−1.5	3.8	3.2	3.7

## 4. Discussion

Modifications to the light-harvesting complex have previously been shown to affect thylakoid membrane organization [11], increase photosystem segregation, and alter photosystem stoichiometry in the set of phycobilisome mutants studied here [25]. We also observed decreased growth rates and biomass accumulation as a consequence of antenna truncation, in particular when cells were grown under specific wavelengths of blue (455 nm) and red (627 nm) light [12]. Recent studies of phycobilisome antenna mutants have continued to explore the relationship between antenna size and productivity under different conditions [26,27,28]. Taken together, our previous studies on these antenna truncation mutants suggested that there are global changes occurring upon antenna modification and their relevance to photosystems prompted the analyses of photosystem function in these mutants.

Low-temperature fluorescence (Figure 1), immunoblot detection of PSI and PSII (Figure 2), PSI quantitation (Figure 3B) and PSII-mediated oxygen evolution (Figure 4) all demonstrated that an important physiological response to decreasing PBS antenna size is to modulate photosystem stoichiometry. The increase in PSII expression was expected, as it has been previously reported using a variety of other methods [8,10,21]. A decrease in maximum photo-oxidizable P700 with a decrease in antenna size is indicative of a decrease in active PSI (Figure 3A,B). This decrease is also evident from immunoblots (Figure 2). PBSs have recently been shown to transfer excitation energy to both the photosystems [3]. Since PSII has a much smaller chlorophyll antenna than PSI, PBS depletion has a proportionally greater effect on PSII antenna size compared to PSI. A decrease in PSI with a consequent increase in PSII titers might therefore be essential to ensure optimal energy delivery to PSII in antenna mutants.

The need to upregulate PSII was also apparent from flash-induced oxygen evolution (Figure 5). During flash-induced oxygen evolution, an increase in damping occurs from the onset of antenna truncation, where CB and CK exhibited a much higher rate of damping than WT. PAL was affected even more significantly, as it only completed two complete turnovers of the S-state cycle before the oxygen evolution signal was completely damped out (WT had completed five turnovers when the measurement stopped). Further analysis of these data showed that the reason behind this increase in damping was a decrease in single hits, with a concomitant increase in misses (Table 1). With decreased antenna size, an increase in misses is to be expected, as pigment number per antenna also decreases.

There is an important correlation between the miss rates in the flash-induced oxygen evolution (Table 1) and in the PSII-mediated oxygen evolution (Figure 4). In Figure 4, at the highest light intensity (8250 µmol photons m^−2^·s^−1^), it appears that CK and PAL had similar rates of oxygen evolution. In Table 1, the single hit rates are similar in this regard, with WT and CB forming a group and CK and PAL forming a separate and distinct group. The reason for this is presumably the drastic increase in misses that occurs in CK compared to CB. This is also apparent from the increase in V_max_ in CK (Table 1). The fact that CB shows minor deviation from WT, while there is a significant change between CB and CK in most data, indicated that there are significant changes that occur with the complete removal of PC rods. The increase in misses during the S-state cycle in CK and PAL, in addition to the increase in PSII titer, resulted in an increase in V_max_ of steady state oxygen evolution. Between WT and CB, there were differences in the PSII-mediated oxygen evolution, in that CB saturated at a higher light intensity. This was evident from the large increase in K_m_ (Table 1), which indicated a loss of ability for light capture due to the truncation of PC rods. The effect is amplified with the severity of the truncation, as observed with CK and PAL.

PSI, quantified based on maximum oxidized P700, showed a decrease consistent with a decrease in antenna size. In this case, CB and CK had a similar concentration of PSI. This observation was different from the trend in which WT and CB were more similar when compared to CK and PAL (Figure 2). However, analysis of the kinetics of P700 oxidation in CB and CK showed significant differences that highlighted changes occurring with the complete removal of rods. A reduction of P700^+^ observed in WT and CB is absent in CK and PAL (Figure 3B). This indicates that complete removal of rods, as observed in CK and PAL, led to an inefficient reduction of the PQ pool. PSII-mediated oxygen evolution measurements showed that optimal electron transfer occurs within PSII (Figure 4). Therefore, an absence of the transient reduction in CK and PAL suggested a slow transfer of electrons from PSII to PSI. This is also evident from the slow decay of P700^+^ observed in PAL mutants after the light is switched off. This effect might indicate that Cyt *b_6_f*, which shuttles electrons between PSII and PSI, was also affected. Removal of rods and core, as seen in PAL, caused a slow reduction of P700^+^. This could be due to the difficulty in carbon fixation, which would explain the inability of PAL to grow at higher pH [12]. Alternatively, it is possible that the overall rate of electrons from PSII is lower in the PAL and CK mutants under the given conditions of illumination.

This study highlights the changes that occur during the transition from WT phycobilisomes to removal of only proximal rods and complete removal of rods and core. This is noticeable with WT and CB being grouped together in PSII-mediated oxygen evolution rates and S-state transitions. CB and CK were similar with respect to changes in photosystem stoichiometry and the affinity for light capture estimated as a measure of K_m_. Significant change in transition from CB to CK is also apparent from the electron micrographs of WT and the three antenna mutants. A visible change is observed in thylakoid membrane curvature of CK and PAL that is not seen in WT and CB [11]. The proximal rod hexamer has been found to prevent proteolysis of Ferredoxin-NADP^+^ reductase protein in cyanobacteria [29]. Therefore, removal of the proximal rod hexamer, but not the two distal rods, could be predicted to have a large effect on substrate channeling and the natural physiology of the organism. The overall photosynthetic capacity is diminished in antenna mutants, as observed in previous studies [12,22]. However, the possibility that these mutants may perform slightly better at higher light intensities cannot be ignored. Recent studies have investigated other conditions that might be beneficial for improved productivity and use in biotech applications [26,27,28].

The removal of the proximal rod hexamer in the transition from CB to CK has major physiological consequences for *Synechocystis* 6803. The differences were apparent in low-temperature (77K) emission spectra when excited for PBSs (Figure 1B). The lack of PC rods also led to an inefficient reduction of PQ pool observed in P700 oxidation kinetics (Figure 3B inset) and the concomitant increase in misses during the S-state transitions (Figure 4, Table 1). All of these changes resulted in a drastic increase in the amount of PSII expressed in CK and PAL, which represents a significant metabolic burden on these cells. Therefore, removal of the proximal PC hexamer alters photosystem functions, which in turn affect the redox balance, causing global effects on the physiology of *Synechocystis* 6803.

## 5. Conclusions

A gradual decrease in the size of light-harvesting antenna leads to changes in photosystem function. Changes in PSII and PSI stoichiometry to increase the PSII to PSI ratio is accompanied by increase in PSII activity. However, there is a decrease in the electron transfer rates from PSII to PSI in antenna mutants that leads to an overall decrease in photosynthetic capacity. Removal of phycocyanin rods and further removal of core also changes the physiology of the organism by affecting the photosystem function, redox balance and thylakoid membrane organization in addition to the light-harvesting efficiencies.

## References

[1] Glazer A.N. (1989). Light guides—Directional energy-transfer in a photosynthetic antenna. J. Biol. Chem..

[2] Kaňa R., Kotabová E., Komárek O., Šedivá B., Papageorgiou G.C., Prášil O. (2012). The slow S to M fluorescence rise in cyanobacteria is due to a State 2 to State 1 transition. Biochim. Biophys. Acta.

[3] Liu H., Zhang H., Niedzwiedzki D.M., Prado M., He G., Gross M.L., Blankenship R.E. (2013). Phycobilisomes supply excitations to both photosystems in a megacomplex in cyanobacteria. Science.

[4] Melis A. (2009). Solar energy conversion efficiencies in photosynthesis: Minimizing the chlorophyll antennae to maximize efficiency. Plant Sci..

[5] Ort D.R., Melis A. (2011). Optimizing antenna size to maximize photosynthetic efficiency. Plant Physiol..

[6] Elmorjani K., Thomas J.C., Sebban P. (1986). Phycobilisomes of wild-type and pigment mutants of the cyanobacterium *Synechocystis* PCC 6803. Arch. Microbiol..

[7] MacColl R. (1998). Cyanobacterial phycobilisomes. J. Struct. Biol..

[8] Ajlani G., Vernotte C. (1998). Construction and characterization of a phycobiliprotein-less mutant of *Synechocystis* sp. PCC 6803. Plant Mol. Biol..

[9] Ughy B., Ajlani G. (2004). Phycobilisome rod mutants in *Synechocystis* sp. strain PCC 6803. Microbiology.

[10] Collins A.M., Liberton M., Jones H.D., Garcia O.F., Pakrasi H.B., Timlin J.A. (2012). Photosynthetic pigment localization and thylakoid membrane morphology are altered in *Synechocystis* 6803 phycobilisome mutants. Plant Physiol..

[11] Liberton M., Page L.E., O’Dell W.B., O’Neill H., Mamontov E., Urban V.S., Pakrasi H.B. (2013). Organization and flexibility of cyanobacterial thylakoid membranes examined by neutron scattering. J. Biol. Chem..

[12] Page L.E., Liberton M., Pakrasi H.B. (2012). Reduction of photoautotrophic productivity in the cyanobacterium *Synechocystis* sp. strain PCC 6803 by phycobilisome antenna truncation. Appl. Environ. Microbiol.

[13] Allen M.M. (1968). Simple conditions for growth of unicellular blue-green algae on plates. J. Phycol..

[14] Porra R.J., Thompson W.A., Kriedemann P.E. (1989). Determination of accurate extinction coefficients and simultaneous-equations for assaying chlorophyll-*a* and chlorophyll-*b* extracted with 4 different solvents—Verification of the concentration of chlorophyll standards by atomic-absorption spectroscopy. Biochim. Biophys. Acta.

[15] Kashino Y., Lauber W.M., Carroll J.A., Wang Q., Whitmarsh J., Satoh K., Pakrasi H.B. (2002). Proteomic analysis of a highly active photosystem II preparation from the cyanobacterium *Synechocystis* sp. PCC 6803 reveals the presence of novel polypeptides. Biochemistry.

[16] Sonoike K., Katoh S. (1989). Simple estimation of the differential absorption coefficient of P700 in detergent-treated preparations. Biochim. Biophys. Acta.

[17] Mannan R.M., Pakrasi H.B. (1993). Dark heterotrophic growth-conditions result in an increase in the content of photosystem II units in the filamentous cyanobacterium *Anabaena variabilis* ATCC 29413. Plant Physiol..

[18] Norling B., Zak E., Andersson B., Pakrasi H. (1998). 2D-isolation of pure plasma and thylakoid membranes from the cyanobacterium *Synechocystis* sp. PCC 6803. FEBS Lett..

[19] Bricker T.M., Lowrance J., Sutton H., Frankel L.K. (2001). Alterations of the oxygen-evolving apparatus in a (448)Arg > (448)S mutant in the CP47 protein of photosystem II under normal and low chloride conditions. Biochemistry.

[20] Meunier P.C. (1993). Oxygen evolution by photosystem II—The contribution of backward transitions to the anomalous behavior of double-hits revealed by a new analysis method. Photosynth. Res..

[21] Stadnichuk I., Lukashev E., Elanskaya I. (2009). Fluorescence changes accompanying short-term light adaptations in photosystem I and photosystem II of the cyanobacterium *Synechocystis* sp. PCC 6803 and phycobiliprotein-impaired mutants: State 1/State 2 transitions and carotenoid-induced quenching of phycobilisomes. Photosynth. Res..

[22] Zhang P., Frankel L.K., Bricker T.M. (2014). Integration of apo-α-phycocyanin into phycobilisomes and its association with FNR_L_ in the absence of the phycocyanin α-subunit lyase (CpcF) in *Synechocystis* sp. PCC 6803. PLoS One.

[23] Joliot P., Barbieri G., Chabaud R. (1969). Model of the system II photochemical centers. Photochem. Photobiol..

[24] Kok B., Forbush B., McGloin M. (1970). Cooperation of charges in photosynthetic O_2_ evolution-I. A linear four step mechanism. Photochem. Photobiol..

[25] Liberton M., Collins A.M., Page L.E., O’Dell W.B., O’Neill H., Urban V.S., Timlin J.A., Pakrasi H.B. (2013). Probing the consequences of antenna modification in cyanobacteria. Photosynth. Res..

[26] Lea-Smith D.J., Bombelli P., Dennis J.S., Scott S.A., Smith A.G., Howe C.J. (2014). Phycobilisome-deficient strains of *Synechocystis* sp. PCC 6803 have reduced size and require carbon-limiting conditions to exhibit enhanced productivity. Plant Physiol..

[27] Kirst H., Formighieri C., Melis A. (2014). Maximizing photosynthetic efficiency and culture productivity in cyanobacteria upon minimizing the phycobilisome light-harvesting antenna size. Biochim. Biophys. Acta.

[28] Kwon J.-H., Bernát G., Wagner H., Rögner M., Rexroth S. (2013). Reduced light-harvesting antenna: Consequences on cyanobacterial metabolism and photosynthetic productivity. Algal Res..

[29] Thomas J.C., Ughy B., Lagoutte B., Ajlani G. (2006). A second isoform of the ferredoxin:NADP oxidoreductase generated by an in-frame initiation of translation. Proc. Natl. Acad. Sci. USA.

